# Evaluation of elevated serum apelin-13 and D-dimer concentrations in individuals diagnosed with pulmonary embolism

**DOI:** 10.1186/s12245-024-00619-z

**Published:** 2024-04-02

**Authors:** Alireza Mehrban, Fatemeh Ahmadi Hajikolaei, Mehdi Karimi, Reza Khademi, Akram Ansari, Durdi Qujeq, Karimollah Hajian-Tilaki, Mahmood Monadi

**Affiliations:** 1 Shariati Hospital, Tehran University of Medical Sciences (TUMS), Tehran, Iran; 2 Babol University of Medical Sciences (MUBabol), Babol, Iran; 3 Bogomolets National Medical University (NMU), Kyiv, Ukraine; 4 Student Research Committee, Faculty of Medicine, Mashhad University of Medical (MUMS) , Mashhad, Iran; 5 Shantou University Medical College, Shantou, Guangdong China; 6 Department of Clinical Biochemistry, Babol University of Medical Sciences (MUBabol), Babol, Iran; 7 Department of Social Medicine, Faculty of Medicine, Babol University of Medical Sciences (MUBabol), Babol, Iran; 8 Department of Internal Medicine, Babol University of Medical Sciences (MUBabol), Babol, Iran; 9 School of Medicine, Babol University of Medical Sciences (MUBabol), Babol, Iran

**Keywords:** Pulmonary embolism, Thromboembolism, Apelin-13, D-dimer

## Abstract

**Background:**

Given the limited specificity of D-dimer, there is a perceived need to discover a more precise marker for diagnosing individuals who are suspected of having pulmonary embolism (PE). In this study, by evaluating the increase in the serum level of Apelin-13 and D-dimer, we found valuable findings about Apelin-13, which can be suggested as an auxiliary and non-invasive diagnostic biomarker in individuals with suspected PE, based on the obtained results.

**Methods:**

In this case-control study, 52 Iranian individuals were included, all of whom were suspected to have PE. These individuals were then divided into two groups based on the results of CT angiography, which is considered the gold standard imaging method for diagnosing PE. The two groups were patients with PE and patients without PE. Finally, the levels of certain markers in the serum were compared between the two groups.

**Results:**

The mean serum D-dimer levels in patients with PE were significantly elevated (*p* < 0.001) in comparison to those without PE (1102.47 to 456.2 ng/ml). Furthermore, the mean level of Apelin-13 was significantly higher in patients with PE (49.8 to 73.11 ng/L) (*p* < 0.001). The cutoff point of Apelin-13 has been calculated at 58.50 ng/ml, with 90.9% sensitivity and 90% specificity. The D-dimer cutoff point was 500 ng/ml, with 95.5% sensitivity and 43.3% specificity.

**Conclusions:**

Based on the results of this study, the serum level of Apelin-13 can be used as a novel diagnostic and screening biomarker in patients with pulmonary thromboembolism.

## Introduction

Pulmonary thromboembolism, commonly known as pulmonary embolism (PE), is a clinical and pathophysiological syndrome arising from the obstruction of a pulmonary artery or its branches by emboli originating from the venous system or right heart. This obstruction leads to dysfunction in the pulmonary circulation and respiratory system [1, 2]. PE stands as the third most prevalent cardiovascular condition, following coronary artery disease and stroke [3]. Individuals diagnosed with PE and categorized as high-risk encountered a mortality rate of 25% [4]. Diagnosing PE is challenging due to the presence of nonspecific signs, symptoms, and associated risk factors [5]. The gold standard for identifying the occurrence of an acute PE is computed tomography pulmonary angiography (CTPA). This method, known for its sensitivity and specificity, not only confirms the existence of a PE but also evaluates the extent and severity of the anatomical clot burden. The decision to employ contrast and undergo radiation exposure in the assessment of a potential PE is a common dilemma in emergency medicine. The legitimate concern lies in the radiation load, and it’s worth noting that CTPA is not recommended for individuals in the low-risk category, especially those with a negative D-dimer result [6].

D-dimer is a product released during the coagulation and breakdown of blood clots, through the degradation of cross-linked fibrin. In patients suspected of PE, the elevation levels of D-dimer in the plasma indicate the likelihood of PE [7, 8]. Highly sensitive plasma D-dimer tests can reliably rule out PE in individuals with a low or intermediate pretest probability of PE [9, 10]. Apelin is a newly discovered endogenous ligand of the G-protein-coupled receptor APJ. It can hydrolyze into several subtypes, the most active of which is Apelin-13. Apelin-13 is an adipokine that regulates a variety of biological processes, including oxidative stress, inflammation, apoptosis, and energy metabolism [11].

Several studies have reported elevated serum levels of Apelin-13 in individuals with venous thromboembolism, suggesting its involvement in the prothrombotic cascade. Notably, individuals with acute respiratory distress syndrome (ARDS) display a substantial increase in Apelin levels, observed in both lung tissue and plasma [12]. On the contrary, individuals diagnosed with pulmonary arterial hypertension (PAH) demonstrate a decrease in Apelin levels [13]. Hemodialysis patients with PAH experience notably lower serum apelin levels compared to those with normal arterial pressure, and this disparity remains unaffected by hemodialysis [14]. It’s worth noting that approximately one-third of patients experiencing symptoms of venous thromboembolism (VTE) are identified with PE, while the remaining two-thirds receive a diagnosis of isolated deep vein thrombosis (DVT) [15] D-dimer shows a lack of precision in identifying VTE, particularly in elderly patients with notable coexisting conditions including infection, syncope, heart failure, trauma, and malignancies [16]. These findings suggest the participation of the apelin/APJ pathway in the onset of respiratory conditions.

This study aimed to evaluate the elevation serum concentrations of both Apelin-13 and D-dimer in individuals suspected of having PE within the Iranian population, to identify a more precise diagnostic biomarker.

## Materials and methods

### Study design

This study was conducted at Rouhani Hospital in Babol, Iran between September 2018 and April 2019. The aim was to investigate the serum levels of Apelin-13 and D-dimer in 52 inpatients suspected of PE within 6 months. Patients with symptoms suggestive of PE were eligible for inclusion. Exclusion criteria included malignant diseases, recurrence of PE, use of drugs that affect the level of D-dimer and Apelin-13, such as long-term use of warfarin and other anticoagulant drugs, and a history of PE. The study was approved by the ethics committee of Babol University of Medical Sciences (IR.MUBABOL.HRI.REC.1397.272), and all patients provided written informed consent to participate in the trial.

### Study procedure

The study included 52 individuals with potential diagnoses of PE based on their clinical symptoms, as determined by the Wells score. The Wells score is a clinical prediction tool employed to categorize patients suspected of having PE. Physicians utilized the seven-item Wells score, which ranges from 0 to 12.5 (higher scores indicating an increased probability of PE), to assess the patient’s clinical pretest probability (C-PTP). A Wells score of 0 to 4.0 was categorized as low C-PTP, 4.5 to 6.0 as moderate C-PTP, and 6.5 or higher as high C-PTP. All the Individuals exhibiting a high clinical pretest probability (C-PTP) underwent chest imaging (CT angiography of pulmonary). Patients with chest imaging findings suggestive of PE received anticoagulant therapy; otherwise, anticoagulant therapy was not performed for other patients. In all the inpatients, venous blood samples were collected within the initial 6 h of admission and before beginning anticoagulation to check the serum levels of Apelin-13 and D-dimer. In this case-control study, based on CT angiography findings, as a diagnostic gold standard, 52 Participants were segregated into two groups based on the presence or absence of PE. In the end, the serum levels of these markers were compared in two groups.

### CTPA Assessment

All CTPA examinations were carried out in a 64-slice CT scanner. In adherence to the standard CTPA protocol, an intravenous contrast agent of 80–100 mL, with an iodine concentration of 350 mg/mL, was administered to all patients. Two senior radiologists investigated all CTPA images. The examination reported the existence of any intraluminal filling defect, along with its location characterized by central or peripheral, and unilateral or bilateral distribution within the pulmonary arterial system and its branches down to a sub-segmental level. Central PE was defined as the presence of an embolus within the right or left main pulmonary artery. Lobar PE was identified when an embolus was present in the right or left lobar pulmonary arteries. Peripheral PE was described as the presence of an embolus in the segmental and sub-segmental arteries.

### Serum apelin-13 concentrations determination by ELISA

To check the markers, 3–5 ml of blood sample was taken from each patient. The amount of D-dimer and Aplin-13 by ELISA (Enzyme-Linked Immunosorbent Assay) was measured. Due to measure Apelin-13, serum was separated from the blood cells using centrifugation for 10 min at 5000 g and kept frozen at -80 °C till the analysis was performed. The amount of Apelin-13 was detected using an ELISA kit. The kit is available from Eastbiopharm under catalog number CK-E11153. The sensitivity of this method is 0.27 ng/L and this method is quantitative (Assay range of 0.5 to 200 ng/ml). Assays were carried out according to the kit instructions.

### Statistical analysis

The sample size was calculated as all patients according to their symptoms who had a possible diagnosis of PE, were included in the study during the 6 months, based on the inclusion and exclusion criteria. The analysis of the data was conducted utilizing The Statistical Package of Social Science Software (SPSS version 18). The variables with normal distribution were reported as percentages, mean values, and standard deviation. Variables without normal distribution were reported as median with range. The chi-square test and t-test were utilized for the comparison of proportions. The Mann-Whitney U test was utilized for non-normally distributed variables to compare the two groups. Receiver Operating Characteristic (ROC) analysis was employed to establish cutoff points and examine the sensitivity and specificity. The overall ROC curve performance was quantified using computing AUC (area under the curve). A p-value less than 0.05 was deemed statistically significant in all comparisons.

## Results

**Fig. 1:**
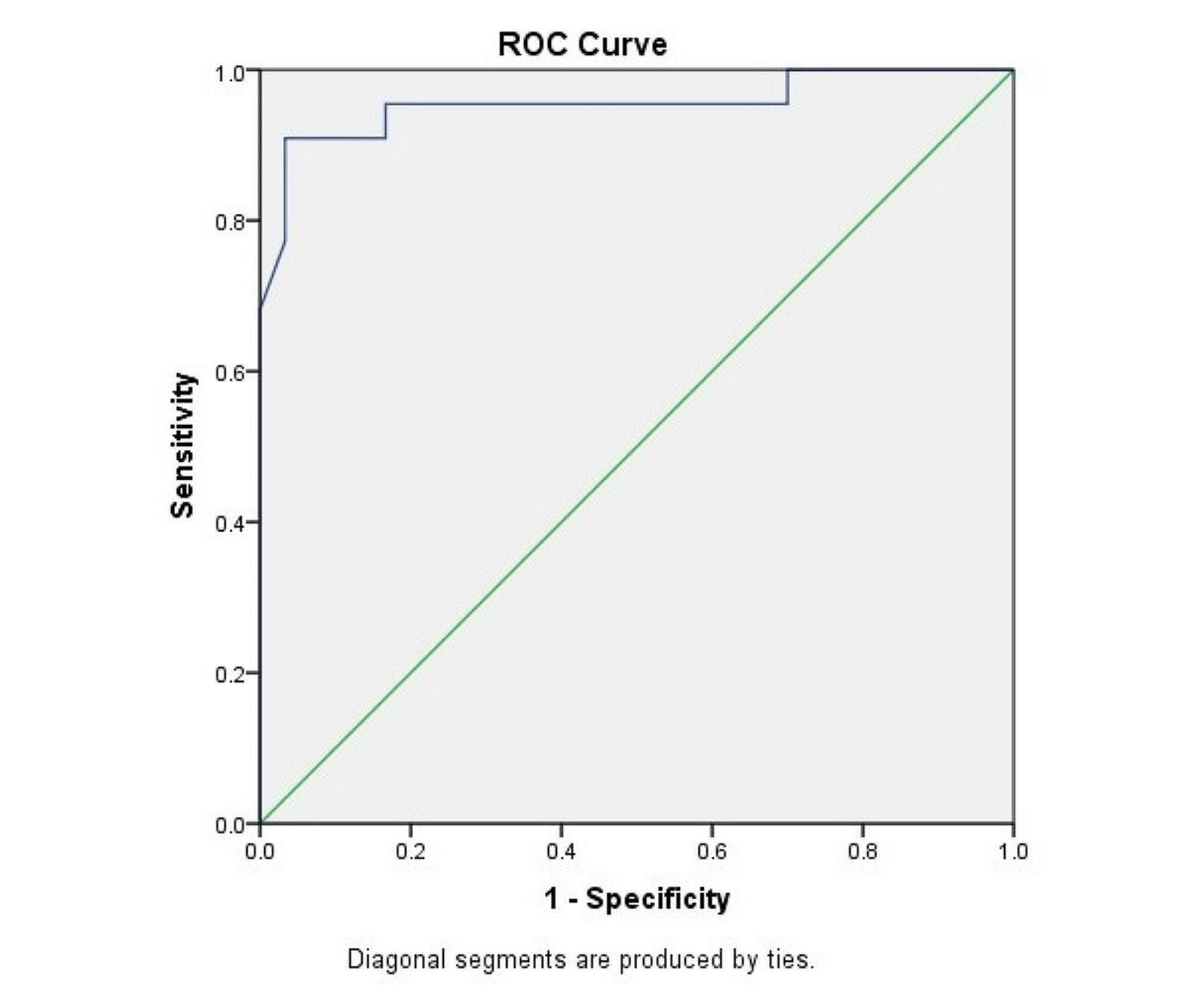
; ROC curve showing the relationship between the specificity and sensitivity of Apelin-13 in the diagnosis of PE

In this study, sampling was available and 52 inpatients in Rohani Hospital in Babol for 6 months with symptoms suspected of PE, they included in this study. Based on the CT angiography finding as the diagnostic gold standard, 22 patients (42.3%) were with PE and 30 patients (57.7%) were without PE. Of these, 23 (44.2%) men and 29 (55.8%) women entered in this study. The mean age of the patients with and without PE was 63.67 ± 10.00 years, ranging from 46 to 86 years. In the examination of the D-dimer marker without taking embolism into account in the surveyed individuals, 14 patients (26.9%) had a normal D-dimer and 38 patients (73.1%) had an increased D-dimer level. In the investigation of the Apelin-13 marker without considering embolism in the studied patients, based on the calculated cutoff point, 29 patients (55.8%) had the Apelin-13 level below the cutoff point and 23 patients (44.2%) had the Apelin-13 level above the cut-off point. The average level of D-dimer in all patients was equal to 729.85 ± 90.471 ng/ml (minimum 107 and maximum 2150) and the average level of Apelin-13 was equal to 59.50 ± 15.15 ng/ml (minimum 28 and maximum 94). The age and gender variables showed no significant difference between patients with PE and those without it (*p* = 0.34 and *p* = 0.68, respectively). On the other hand, among the 52 patients suspected of PE who were investigated, 22 participants (42.3%) had PE, and patients in the without PE group including 13 patients (25.0%) had aggravated COPD, 9 patients (17.3%) had pulmonary edema, 5 patients (9.6%) had aggravated asthma, and 3 people (5.8%) had angina. The average D-dimer level in patients with PE (1102.47 ± 55.16) was significantly higher than in those without PE (456.20 ± 53.36), and this difference was found to be statistically significant (*p* < 0.001). Additionally, the mean Apelin-13 level was significantly elevated in the PE group compared to the non-PE group (73.11 vs. 49.8 ng/ml, *p* < 0.001). According to the ROC curve analysis, the Apelin-13 cut-off point was determined to be 50.58 ng/ml, with a reported sensitivity of 90.9% and specificity of 90% (Fig. 1).

Among the 22 patients with PE, 20 patients (90.9%) had Apelin-13 levels of more than 58.50 ng/ml which was significant (*p* < 0.001). The D-dimer cutoff point is considered equal to 500 ng/ml [17], and based on this cutoff point, the sensitivity is 95% with a confidence interval of 87–100% and the specificity of 43% with a confidence interval of 26–61% was reported. A D-dimer level exceeding 500 ng/ml was observed in 21 patients (95.5%) within the PE group with *p* = 0.002 which concluded the relationship was significant. In the evaluation of the relationship between Apelin-13 level and having PE according to Tables 1 and 2, it can be stated that in 20 patients (90.9%) with PE, the level of Apelin-13 has increased the difference was reported significant (*p* < 0.001). Table 3 shows that in either women or men patients, the average D-dimer level in individuals with PE was significantly higher than that in others without it and the difference reached statistical significance (*p* = 0.001 and *p* = 0.001, respectively). Both in women and men, the mean Apelin-13 level was significantly higher in individuals with PE compared to those without it, with statistical significance observed (*p* < 0.001).

**Table 1:** Quantitative comparison of D-dimer and apelin-13 levels in patients in two groups by age group

	Age	PE group (mean ± SD)	No PE groups (mean ± SD)	P value
D-dimer (ng/ml)	< 60	715.143 (±17.82)	367.177 (±33.38)	0.002
	≥ 61	1247.48 (±81.27)	516.210 (±00.58)	< 0.001
Apelin-13 (ng/ml)	< 60	71.12 (±83.60)	50.6 (±42.21)	< 0.001
	≥ 61	73.10 (±69.92)	48.9 (±83.49)	< 0.001

**Table 2:** Comparison of patients in case-control groups based on the cutoff points of Apelin-13 and D-dimer

Groups	Apelin-13	D-dimer		P value
		Positive (n)	Negative (n)	
Without PE	Positive (n)	3	-	0.23
	Negative (n)	14	13	
PE	Positive (n)	20	-	0.09
	Negative (n)	1	1	

**Table 3:** Quantitative comparison of D-dimer and apelin-13 levels in patients in two groups by gender

	Gender	PE group (mean ± SD)	non-PE group (mean ± SD)	P value
D-dimer (ng/ml)	Male	1379.526 (±67.76)	558.172 (±86.25)	0.001
	Female	910.343 (±69.01)	367.199 (±00.76)	0.001
Apelin-13 (ng/ml)	Male	75.14 (±11.19)	50.5 (±36.94)	< 0.001
	Female	71.8 (±85.82)	48.9 (±69.98)	< 0.001

The risk of VTE and PE increases with age. According to certain studies, both men and women see an increase in the incidence of VTE and PE after the age of 60, with PE accounting for the majority of the increase [18].

The current study shows that in both individuals aged below 60 and those aged 61 years or older, the mean level of D-dimer in patients with PE is notably elevated compared to patients without PE, and this disparity is significant (*p* = 0.002 and *p* < 0.001, respectively). In both age groups—those under 60 years old and those aged 61 years or older—patients with PE exhibited a significantly higher average level of Apelin-13 compared to those without it, with significant differences (*p* < 0.001). Among the male patients with PE, 88.9% showed a significant increase in Apelin-13 levels (*p* < 0.001), while in female patients with PE, 84.6% exhibited a significant elevation in Apelin-13 levels (*p* < 0.001). Based on the results of Table 2 show that 20 patients with PE (90.9%) had serum levels of D-dimer and Aplin-13 were reported positive. However, the relationship between these two markers in patients with PE was not significant (*p* = 0.09). 17 patients without PE had high D-dimer (56.7% false positive and 43.3% specificity) and 3 patients without PE had high Apelin-13 (10% false positive and specificity of 90%). In patients without PE, 13 patients (43%) both D-dimer and Apelin-13 levels were negative and this relationship was not statistically significant (*p* = 0.23). Among individuals with PE, 21 of them had a high D-dimer level (95.5% diagnostic sensitivity) and 20 patients had a high Apelin-13 level (90.9% diagnostic sensitivity) (Table 1).

According to Table 2, the positive predictive values of Apelin-13 and D-dimer were 86.9% and 55.2%, respectively. Furthermore, Apelin-13 and D-dimer demonstrated negative predictive values such as 93.1% and 92.8%, respectively.

## Discussion

This research is among a handful of sporadic studies that have explored the serum concentration of Apelin-13 in individuals with suspected PE. Over the past few years, there has been a growing body of scientific studies delving into the role of Apelin-13 in the pathophysiology of hypoxic conditions [19, 20]. While the impact of hypoxia on the regulation of Apelin-13 in hypoxic conditions in humans should be clarified, several studies have demonstrated that the expression of Apelin-13 in the endothelium, adipocytes, and lung cells of animal samples was enhanced under hypoxic conditions [19, 21, 22]. The lung serves as the primary origin of Apelin-13 receptors in the bloodstream [23]. During the acute phase of PE, numerous vasoactive substances are released, and Apelin-13 might exert an antagonistic influence on vasoconstrictor mechanisms that rely on nitric oxide (NO) [24, 25]. According to several studies, the current D-dimer biomarker, which is used to diagnose acute PE in suspected patients, does not have high specificity. In low-risk populations, the sensitivity of D-dimers is high, but their specificity for detecting DVT or PE is diminished [26].

In this research, it was found that the levels of D-dimer and apelin-13 increased dramatically. Among individuals experiencing PE. The results of our study suggest that Apelin-13 could be viewed as a novel biomarker and a potential focus for therapy in future cases of acute PE. A notable outcome from the current study was the demonstrated high sensitivity and specificity of Apelin-13 at the threshold of 58.50 ng/ml. The 90.9% sensitivity of this marker means that out of every 100 people who have PE according to the gold standard, based on the level of Apelin-13 higher than 58.5 ng/ml, it has correctly diagnosed more than 90 people, which has high sensitivity. Regarding the 90% specificity of this marker, it can be said that out of 100 people who were healthy according to the gold standard, the level of Apelin-13 above 58.5 ng/ml correctly identified 90 people as healthy people, which is much higher than the findings related to D-dimer in the studies.

In a study conducted by Selimoglu et al., a significant increase in the serum level of Apelin-13 was shown in the PE group compared to the control group. They concluded that the level of Apelin-13 increases among individuals diagnosed with PE [27]. This aligns with the outcomes observed in the current study. There is a consensus regarding the increase in D-dimer serum levels with venous thromboembolism, and many studies in this field reported similar results [28]. According to Yoshiiwa et al., examining D-dimer levels proves beneficial for promptly diagnosing thromboembolism. They further highlighted that an elevated D-dimer level is considered a contributing factor for PE [29]. Tang et al. observed that a rise in D-dimer levels correlated with an elevated likelihood of pulmonary thromboembolism [30]. Karataş et al. determined a cutoff point of 1579 ng/ml for Apelin-13 in their study, revealing a sensitivity of 92.7% and a specificity of 96.7%. The difference in the obtained cut point is due to the difference in the Apelin-13 measurement kit [31]. Also, the sensitivity and specificity obtained in the Karataş study are slightly higher than in the present study. However, in both studies, the demonstrated sensitivity and specificity at this level suggest that the serum Apelin-13 level can serve as a novel diagnostic biomarker for individuals with pulmonary thromboembolism. Makris et al. reported that the most specific test to confirm the definitive diagnosis of pulmonary thromboembolism is selective pulmonary angiography, which can also detect emboli as small as 1–2 mm, but this method is invasive and causes possible complications in the patient [32]. Also, although CT angiography has recently been proposed as a diagnostic gold standard in studies [33], for people who have a low susceptibility based on the existing criteria and scores for diagnosis, performing this method is not justified. Therefore, according to the finding of high sensitivity and specificity for Apelin-13 as a less expensive, non-invasive, and uncomplicated diagnostic biomarker, it can be suggested to identify people with pulmonary thromboembolism.

The sensitivity and specificity of D-dimer constitute another aspect of debate within this research at its standard cut point of 500 ng/ml. In the current study, the sensitivity is 95% and the specificity is 43%. At the conventional threshold, Gao et al. documented D-dimer’s sensitivity and specificity as 96.2% and 50%, respectively [34]. With its elevated sensitivity, D-dimer can serve as an effective screening method [26]. Glober et al. observed a sensitivity of 95.7% and a specificity of 40% for D-dimer in diagnosing PE in their study [35]. In a review study, the sensitivity and negative predictive value of D-dimer by ELISA for PE were both 100% [36]. Our study exhibited greater sensitivity compared to the review study, whereas the negative predictive value was inferior to that particular investigation. In the context of our study, the negative predictive values for Apelin-13 and D-dimer tests in PE were found to be 93.1% and 92.8%, respectively. Considering that the negative predictive value shows how likely it is that the person is healthy if the desired test is negative, this index helps us to use these factors for quick and low-cost triage among individuals with suspected PE, and in cases of high serum levels, standard imaging methods were used.

In a review study, Pulivarthi et al., considering the cut-off point of 500 and differences in sensitivity and specificity in different studies, stated that several elements contribute to the variability in sensitivity and specificity of the D-dimer test. These factors encompass the extent of thrombosis and fibrinolytic activity, duration of symptoms, anticoagulant treatment, concurrent surgical or medical conditions, inflammatory diseases, advanced age, pregnancy, postpartum period, history of previous venous thromboembolism (VTE), and the presence of malignancy [37].

In our research, Apelin-13 and D-dimer tests demonstrated positive predictive values of 86.9% and 55.2%, respectively, for PE. Considering that the positive predictive value shows how likely it is that the person is sick if the desired test is positive, this index helps us to make the right decision to choose the diagnostic algorithm, and Apelin-13 can help reject many false positive cases of the testing of D-dimer. It is necessary to explain that the difference in the sensitivity and characteristics of the biomarkers in the present study can rely on diverse factors such as the type of kit used, the type of measuring device, laboratory factors, and the time of measurement of these factors in individuals according to both the disease’s acute and chronic stages or before the onset of the disease. Of course, due to the lack of studies during the disease and the measurement of these factors in the acute phase and before the start of anticoagulants, it can be claimed that this research is one of the first studies conducted in this field.

An important discovery in this study, recognized as a strength, involves the assessment of two biomarkers—D-dimer and Apelin-13—across various age groups and in both male and female populations. In patients experiencing embolism, both biomarkers exhibited a substantial increase in levels for individuals under 60 years and those over 60, a trend observed across both genders. Therefore, the application of this marker in identifying lung diseases, notably pulmonary thromboembolism, facilitates early diagnosis and, consequently, the timely initiation of patient treatment.

## Conclusions

In conclusion, this study adds to the growing body of research examining the serum concentration of Apelin-13 and its potential as a biomarker in individuals with suspected pulmonary embolism (PE). The findings indicate a significant increase in both Apelin-13 and D-dimer levels among individuals experiencing PE, with Apelin-13 showing promising sensitivity and specificity at a threshold of 58.50 ng/ml. Notably, Apelin-13 demonstrated higher sensitivity and specificity compared to D-dimer, suggesting its potential as a novel diagnostic biomarker for PE. Further research in this area is warranted to validate these findings and explore the clinical utility of Apelin-13 as a diagnostic tool for PE, potentially leading to earlier detection and treatment initiation in affected individuals across different age groups and genders.

## Data Availability

No datasets were generated or analysed during the current study.
